# Development of Infant Sitting Postural Control in Three Groups of Infants at Various Risk Levels for Autism Spectrum Disorder

**DOI:** 10.3390/ijerph20021234

**Published:** 2023-01-10

**Authors:** Anastasia Kyvelidou, Shari DeVeney, Dimitrios Katsavelis

**Affiliations:** 1 Physical Therapy Department, Creighton University, Omaha, NE 68178, USA; 2 Department of Special Education and Communication Disorders, University of Nebraska at Omaha, Omaha, NE 68182, USA; 3 Department of Exercise Science and Pre-Health Professions, Creighton University, Omaha, NE 68178, USA

**Keywords:** autism spectrum disorders, infancy, motor skills, communication, premature birth, low birth weight, sitting posture

## Abstract

The purpose of this study was to examine the development of sitting postural control among two groups of infants at elevated risk for autism spectrum disorders (ASD) and a group of infants at typical risk for ASD and its association with cognitive, language and communication skills at a later age. We visited infants in their home environment from the onset of sitting until sitting independence and at 12 and 18 months of age. We collected data on sitting posture (center of pressure), through a portable force platform, as well as communication, cognitive and social behavior assessments at various time points. Our results showed that postural control differences at the onset of sitting, were present among the groups of infants but there were no statistically significant differences among the groups in the development of sitting posture. In addition, there were statistically significant communication differences among the groups and mostly the change in sample entropy in the anterior/posterior direction (posture measure) was significantly correlated with other skills at a later age. This study highlights the importance of investigating multiple at-risk groups to identify unique developmental pathways that may lead to an ASD diagnosis.

## 1. Introduction

The prevalence of autism spectrum disorder (ASD) has increased dramatically in the last decade. Specifically, based on the Centers for Disease Control and Prevention Autism Developmental Disabilities Monitoring about one in 44 children has been identified with ASD [1]. It has been suggested that the increased prevalence of ASD is due to growth in community awareness and international public health efforts, and advancement in case identification and definition [2]. The increasing occurrence of ASD creates an imperative need for clinicians to identify as early as possible ASD related deficits in order for these children to receive access to early intervention services and possibly a greater chance to improve quality of life. It is important to highlight that detecting differences earlier on in development is an extremely difficult task. In fact, these differences may be due to similar characteristics among elevated-risk groups of infants or to differences in developmental patterns that are, to date, scarcely captured by diagnostic tools [3]. It is possible that early and detectable signs of ASD-related deficits may first become noticeable within the development of the motor system, since it may affect object exploration skills, learning, and subsequently cognitive, language, and social development [4]. Most of the studies that examine early motor skill development in infants at elevated risk for ASD rely on subjective measures and scales of motor function, such as the gross and fine motor skill components of the Mullen Scales of Early Learning or the Alberta Infant Motor Scales. Even though, some attempts have been made at developing qualitative tools for the analysis of fine motor skills and ASD diagnosis [5]. In addition, the most widely researched group of elevated-risk infants that has been examined in terms of early signs for ASD has been those with familial risk for ASD. 

### 1.1. Infants at Elevated-Risk for ASD

The baby siblings research consortium study estimated that approximately 18.7% of children with siblings diagnosed with ASD may later develop ASD, while an even greater percentage may develop general motor, social, and communication deficits [6]. Heritability estimates from twin studies provide strong evidence that genetic factors play a critical role in vulnerability to ASD. The majority of the prospective studies that investigate prodromal signs of ASD involve this group of infants that have familial risk for ASD (e.g., brother or sister diagnosed with ASD). However, there is a need to investigate other cohorts of infants that may be at elevated risk for ASD, excluding the familial predisposition (genetic influence).

Infants born prematurely or with low birth weight (LBW) have significant impacts on their health and well-being. Although several studies have focused on specific mental disorders retrospectively, few studies have examined the association between prematurity or LBW in relation to the prevalence of ASD [7]. Recent evidence suggests that premature born or LBW infants present increased prevalence rates of ASD, of the order of 5% [7]. However, others have suggested that infants born preterm or with LBW very often test positive in ASD screeners. Interestingly, when looking at the combination of preterm birth and LBW the incidence of ASD diagnosis increases to 10–20% [8]. Since the focus on early signs of ASD has been in those infants with familial risk for ASD, there is no available information or studies that examine the severity of ASD, similarities in developmental patterns with respect to other elevated-risk groups, or when diagnosis is usually carried out or symptoms emerge in this group. Irrefutably, more research is needed to establish the accurate prevalence rates of ASD in preterm and LBW infants by considering the appropriateness of weight for gestational age. Nevertheless, the addition of this group of infants provides a unique opportunity, as it would allow for the examination of unique developmental trajectories of children with elevated risk for ASD that are not due to their genetic profile.

### 1.2. Motor Deficits in Infants and Children with ASD

Parents of children with ASD have reported movement-related deficits in their children within the first year of life [9]. These studies provided evidence of delays in early motor milestones, such as rolling, sitting, and crawling as well as the presence of movement asymmetries and atypical reflexes [10,11,12]. However, the results are inconclusive and there is a certain recall bias involved in retrospective evaluations of motor development in children with ASD. Another and more reliable way of assessing motor deficits early in life is through a prospective evaluation of infants and toddlers at elevated risk for ASD. A plethora of recent studies have identified gross and fine motor impairments in at-risk siblings as early as 7 months of age. More and more, motor functioning appears to be a reliable predictor of autism diagnosis [13]. Overall, gross motor delays and specifically postural delays have been documented in infants and toddlers at risk for ASD [3,14].

Postural control is essential for the development of reaching for and engaging with objects and participating in social interaction. The first study that examined prospectively posture development in infants at elevated risk for ASD demonstrated that these infants progressed slower in sitting and standing postures and suggested that deficits or delays in posture skills might affect detrimentally other developmental domains [15]. Examining sitting and reaching, Mlincek and colleagues (2022) [14] suggested that infants at elevated risk for ASD may have difficulty in engaging with other behaviors once they are able to sit independently without the use of support from their hands, due to limited processing resources. However, the study by Nickel and colleagues (2013) [15] as well as other above-mentioned studies that investigated gross motor skills have used subjective tools. In our recently published study that investigated infant sitting postural control and its relationship to other developmental domains [16], we found that certain measures of sitting posture at 6 months of age could predict language and visual reception behavior at 12 months of age. This experimental paradigm which examined the center of pressure (CoP) trajectories, offers an additional level of analysis in gross motor skill development. One of the diagnostic criteria for ASD is repetitive and stereotyped movement behaviors. A very limited number of studies have examined stereotyped movements in infancy that have produced inconclusive findings, since they differ in experimental and analytical approaches. Nonlinear analysis of CoP data offers the opportunity to observe regularities of posture, which may be precursors of repetitive behavior in ASD. Collectively, there is a documented need for an objective and quantitative assessment of gross motor development, which would warrant greater confidence in identifying ASD-related motor deficits in infants at elevated risk for ASD. 

Therefore, the purpose of this study was to expand on the findings of the 2021 [16] study and examine the development of sitting postural control and its association with cognitive, language and communication skills at a later age. Specifically, we examined (a) postural control differences during the development of sitting among infants at typical and elevated risk for ASD, (b) postural control differences at the onset of sitting (similarly to the 2021 [16] study), (c) cognitive, language, and communication differences at 12 and 18 months of age and d) the relationship between the change in postural control measures during the development of sitting, with language, communication, and cognitive behaviors at 12 and 18 months of age. We hypothesized that postural control differences at the onset of sitting will not be present among the groups of infants; however, we speculated that there will be differences between the groups in the development of sitting posture. Moreover, we hypothesized that there will be differences among groups in cognitive, language, and communication domains, and the changes in postural control measures during the development of sitting would be correlated with other skills at a later age. 

## 2. Materials and Methods

### 2.1. Participants

Three groups of infants participated in the study (demographics of the groups are presented in Table 1; two elevated-risk groups, which were infants who were siblings of children with ASD (5) and infants born prematurely and with LBW (8). Those two groups are denoted as ASD and LBW, respectively. One group of typical-risk infants with no familial history of ASD, prematurity, or LBW (TD, 24) was also included. A total of 37 infants participated in the study. The inclusion/exclusion criteria are displayed in Table 2. The average gestational age of the LBW group was 33 weeks and 1 day (SD: 23.5 days). Infants were recruited around the age of 3–4 months of age and were recruited from university announcements, posted fliers on social media, and local autism centers. Informed parental written consent as approved by the university of Nebraska Medical Center institutional review board (Omaha, NE, USA) was received from all participants prior to data collection. Attrition for this study was 0%.

### 2.2. Procedures

All data collections took place at the participants’ homes. All infants attended seven data collection sessions (Table 3). These points in development were selected because of the age at which the sitting skill develops. After the first screening, families were contacted every week to assess onset of sitting. The average age of onset of sitting for the three groups of infants did not differ, and it was around 6 months and 10 days. For the purpose of this manuscript, we included only the postural control and not the eye-tracking data. For additional details regarding the study’s methodology including other developmental domains examined and their associated findings, see DeVeney and Kyvelidou (2020) [17] and DeVeney, Kyvelidou, and Mather (2021) [18].

### 2.3. Instrumentation and Measurement

#### 2.3.1. Sitting Posture

Center of Pressure (CoP) data during sitting was collected by means of a Bertec portable force platform (Bertec, BP5046, Columbus, OH, USA). Data acquisition and processing was controlled through the Bertec Digital Acquire software with a sampling frequency of 200 Hz. CoP displacements in both the anterior-posterior (AP) and medial-lateral (ML) sway directions were calculated through the software. Video of each sitting trial was also collected using a laptop camera that was positioned to record the sagittal view of the infant. CoP data were collected for 10 s while the infant attempted to maintain the sitting position. This was repeated until three trials were collected in which the infant did not fall, cry, or excessively move their arms or trunk. 

#### 2.3.2. MacArthur-Bates Communicative Development Inventories (CDIs)

The CDIs: The infant forms words and gestures (age 6–18 months) assesses receptive and expressive vocabulary and acknowledges the use of communicative and symbolic gestures [19]. The CDIs are parent-reported questionnaires in the form of checklists and are norm referenced. The CDIs were administered at 12 and 18 months of age. Specifically, we examined the number of phrases and words understood, number of words produced, and number of early, later, and total gestures. 

#### 2.3.3. Mullen Scales of Early Learning (MSEL)

The MSEL is a standardized, valid, and reliable general developmental measure for ages birth to 68 months and can assess communication delays [20]. It is comprised of specific domains including gross and fine motor function, expressive and receptive language as well as visual reception. Each scale includes interactive tasks that can be completed by the child or may be scored through parent assistance. The Early Learning Composite standard score (calculated from the Visual Reception, Fine Motor, and Receptive and Expressive Language subscale scores) represents the level of infant cognitive behavior and was completed by a trained experimenter; reliability in scoring between experimenters was monitored throughout testing. In the presented study we report the MSEL data at 12 and 18 months of age. 

#### 2.3.4. Communication and Symbolic Behavior Scales Developmental Profile Infant-Toddler Checklist (CSBS DP)

The CSBS DP was administered at 12 and 18 months of age to measure infant communicative behaviors. This norm-referenced measure is based on parent reports [21]. Consisting of 24 items such as eye gaze, gestures, sounds, words, understanding, and play, the CSBS DP is a valid and reliable measure used in infants and toddlers whose functional communication age is between 6 months and 24 months. 

#### 2.3.5. Data Analysis

The CoP data were analyzed as described in Kyvelidou et al. (2021) [16]. Linear measures included the root mean square (RMS) and the range of the CoP trajectory. In addition, the frequency-domain parameters of the median frequency and frequency dispersion were calculated. Median frequency is the frequency below which 50% of total power is present while frequency dispersion is the variability of the spectral frequency distribution [22]. One nonlinear measure was calculated from the CoP path in both the AP and ML directions: Sample Entropy (SampEn). The nonlinear measures are sensitive to the way the CoP path changes over time and reveal the self-initiated strategies used to control postural sway. The SampEn measure was calculated using the algorithm of Richman and Moorman (2000) [23] implemented in MATLAB. This measure estimates the complexity of physiological time series data and essentially quantifies the probability that similar patterns of observation in the CoP path are not followed temporarily by additional similar observations. Lower values indicated lower complexity and repeatability of the CoP signal, while great values indicate greater complexity and less repeatability of the CoP signal.

#### 2.3.6. Statistical Analysis

All statistical analyses were performed with SPSS version 28.0 statistical software program. Due to our inability to make any reasonable estimate of the underlying distribution of data we performed Mann–Whitney U tests to identify group differences for all dependent measures and Kendall’s Tau b correlations to identify relationships between posture variables and communication, language, and cognitive scores. Effect sizes were calculated using eta squared (η^2^). Lastly, we performed stepwise multiple linear regression analysis to investigate the percentage of variance of each of the communication, language, and cognitive scores at a later age could be explained by the change in posture variables during the development of sitting. The alpha level was set at 0.05.

## 3. Results

**Figure 1:**
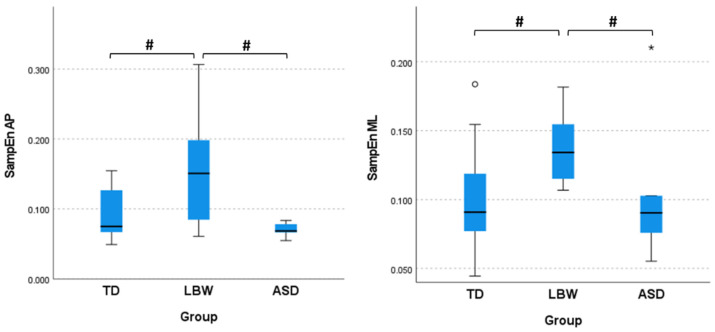
Sample entropy (SampEn) values in the anterior/posterior (AP) and medial/lateral (ML) directions at the onset of sitting posture. TD, infants with typical development; LBW, infants born prematurely and with low birth weight; ASD, infants with a sibling diagnosed with ASD. The # sign indicates significant differences between groups, while the * and ° denote outliers.

The change in sitting postural control parameters revealed no differences among groups. However, when looking only at the onset of sitting (1st session) SampEn in AP (H(2) = 6.239, *p* = 0.044, η^2^ = 0.125) and ML (H(2) = 7.057, *p* = 0.029, η^2^ = 0.149) directions showed statistically significant differences among groups with medium and large effect sizes respectively. The LBW group presented significantly greater SampEn values than both the TD and ASD groups in the AP direction, while in the ML direction, the LBW group presented significantly greater SampEn values than the TD group (Figure 1).

**Figure 2:**
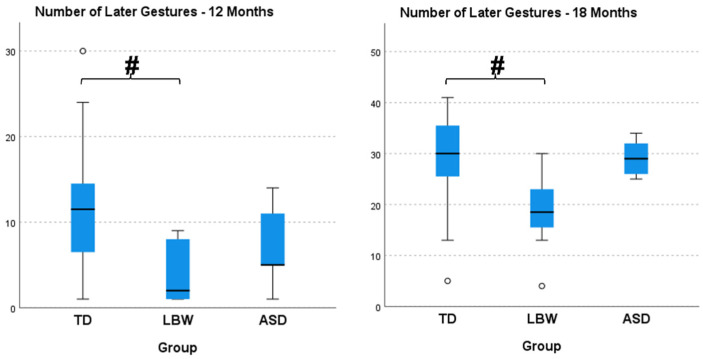
Number of later gestures at 12 and 18 months of age from the MacArthur-Bates Communicative Development Inventories (CDIs). TD, infants with typical development; LBW, infants born prematurely and with low birth weight; ASD, infants with a sibling diagnosed with ASD. The # sign indicates significant differences between groups while the ° denotes outliers.

**Figure 3:**
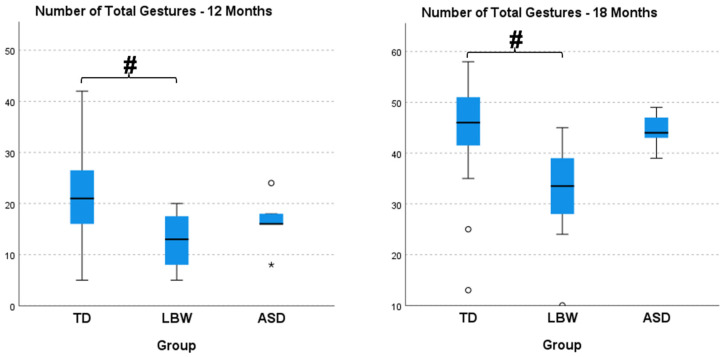
Number of total gestures at 12 and 18 months of age from the MacArthur-Bates Communicative Development Inventories (CDIs). TD, infants with typical development; LBW, infants born prematurely and with low birth weight; ASD, infants with a sibling diagnosed with ASD. The # sign indicates significant differences between groups, while the * and ° denote outliers.

**Figure 4:**
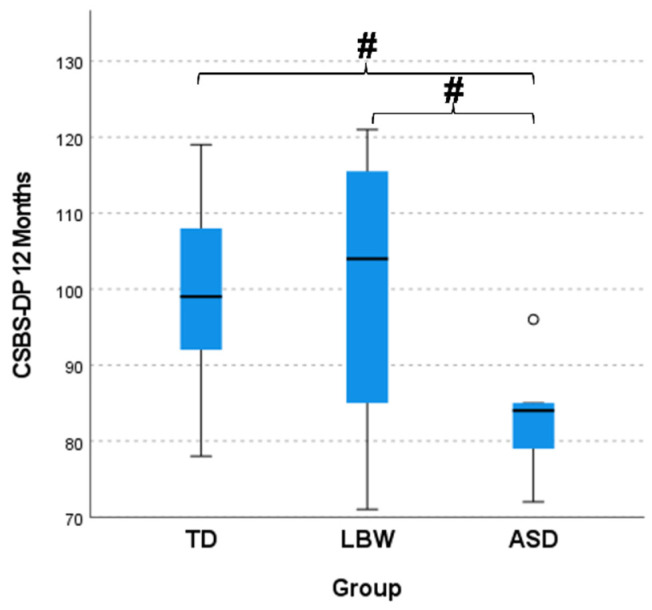
Communication and symbolic behavior scales developmental profile (CSBS-DP) scores at 12 months of age. TD, infants with typical development; LBW, infants born prematurely and with low birth weight; ASD, infants with a sibling diagnosed with ASD. The # sign indicates significant differences between groups, while the ° denote outliers.

At 12 (H(2) = 9.456, *p* = 0.009, η^2^ = 0.219) and 18 (H(2) = 9.455, *p* = 0.009, η^2^ = 0.219) months of age, number of later gestures was significantly different among groups, with large effect sizes. The LBW group presented significantly lower later gestures than the TD group at both 12 and 18 months (Figure 2). At 12 (H(2) = 10.011, *p* = 0.007, η^2^ = 0.236) and 18 (H(2) = 9.730, *p* = 0.008, η^2^ = 0.227) months of age, number of total gestures was significantly different among groups with large effect sizes. The LBW group presented significantly lower total gestures than the TD group at both 12 and 18 months (Figure 3). At 12 (H(2) = 6.342, *p* = 0.042, η^2^ = 0.128) months of age, CSBS-DP was significantly different among groups with medium effect size, with the ASD group showing significantly lower values than both the TD and LBW group (Figure 4). At 18 months of age, there were no differences among the groups in CSBS-DP. 

Stepwise regression analysis was used to capture the proportion of variance that the change in posture variables from the onset of sitting up to sitting independence could predict cognitive and communication behavior at 12 and 18 months. The change in SampEn in the AP direction explained around 32% of the variance in CSBS-DP at 12 months(F(1,35) = 16.793, *p* < 0.001), and around 17% (F(1,35) = 7.096, *p* = 0.012) at 18 months. Infants that presented with decreased SampEn in AP direction during the development of sitting showed greater CSBS-DP values at both 12 and 18 months of age. The change in SampEn in the AP direction explained around 15% of the variance in total (F(1,35) = 6.169, *p* < 0.018) and composite (F(1,35) = 6.207, *p* < 0.018) score in the MSEL at 12 months of age. Infants that presented with decreased SampEn in the AP direction during the development of sitting showed greater total and composite scores in the MSEL at 12 months of age. The change in SampEn in the AP direction (F(1,35) = 7.870, *p* < 0.008) explained around 19% of the variance in number of phrases understood at 12 months, while the change in RMS in the ML direction (F(1,35) = 5.754, *p* < 0.022) explained around 14% of the variance in number of words understood at 12 months. Infants that presented decreased SampEn in the AP direction during the development of sitting showed greater scores in the number of phrases understood at 12 months of age, whereas infants that presented increased RMS in the ML direction during the development of sitting showed great scores in the number of words understood at 12 months of age. 

## 4. Discussion

Our hypotheses were partially verified. Postural control differences at the onset of sitting were present among the groups of infants but there were no statistically significant differences among the groups in the development of sitting posture. In addition, there were statistically significant communication differences among the groups and mostly the change in SampEn in the AP direction (posture measure) was significantly correlated with other skills at a later age. 

Even though we did not expect to observe postural control differences at the onset of sitting, SampEn in the AP direction was significantly greater in the LBW group in comparison to the TD and ASD groups, and in the ML direction, SampEn was significantly greater than the TD group. No differences were observed between ASD and TD groups. In the study by Kyvelidou et al. (2021) [16], SampEn in the ML direction almost reached statistical significance between the ASD and TD groups. One explanation could be the limitation in the sample size of the elevated-risk groups, even though the current results presented moderate to high effect sizes. Conversely, the LBW group might demonstrate greater disturbances in the motor system than the ASD group. The greater values in SampEn in both the AP and ML directions suggest that the postural sway of LBW infants is characterized by increased stochastic components in comparison to infants with TD and ASD. Older children with motor impairments have demonstrated increased postural sway variability with other nonlinear measures of CoP [24]. In contrast, there were no differences observed in the development of sitting postural control when examining the change in postural control variables from onset of sitting up to sitting independence. It is possible, that this approach in examining change (taking the difference between the first and last session of sitting) might have masked the intriguing changes happening during the development of sitting. Thus, a future approach may be more successful if it examines the developmental trajectories of postural control variables across the development of sitting. 

When examining communication and cognitive differences at 12 and 18 months of age, we observed only communication differences among the groups, especially in the number of total and later gestures from the CDIs as well the total standard score from the CSBS-DP. Interestingly, LBW infants presented the lowest scores on the CDIs while the ASD infants presented the lowest scores on the CSBS-DP. In our previous study, we did not observe any differences at 12 months in CSBS-DP scores between the TD and ASD groups, possibly attesting to the heterogeneity of the group of elevated-risk infants. It is possible that the motor impairment level in the LBW group of infants influenced communication gesturing while this was not evident in the ASD group. Conversely, it could also indicate that deficits in gesturing may be subtler in the ASD group or emerges at different time points. Gesturing and fine motor skills have been investigated by others but direct comparisons cannot be made since we were not able to collect later group outcomes in terms of ASD diagnosis (see references [5,25]). Moreover, the CSBS-DP captures social, speech, and symbolic behaviors, which may be more indicative of the deficits that are prominent in the ASD group. Overall, lower scores of infants at elevated risk for ASD have been observed in language, communication, and social behavior domains at 12 months in multiple studies [3,26,27]. 

Even though the change in SampEn in AP did not show differences among the groups of infants, it demonstrated that it could predict between 15–35% of the variance in language, communication, and cognitive behaviors at 12 and 18 months of age. Even though direct comparisons with other studies in the literature are not available, Kyvelidou et al., (2021) [16] showed that measures of postural sway can predict social, cognitive, language, and communication behaviors at 12 months of age. The present study adds concrete and qualitative evidence that changes in postural control during the development of sitting are associated with other developmental domains at a later age (12 and 18 months of age) and corroborate the importance of motor skills and their cascading effect on overall development, particularly their effect on language development [28]. Another important finding is that infants who showed greater scores in the CSBS-DP, MSEL composite score, and some aspects of the CDIs, developed more repeatable patterns in the CoP path in the AP direction, as well as increased the magnitude of the postural sway in the ML direction. It is possible that as infants become more efficient and experienced in the sitting skill, motor responses become more automatic, and in a way, postural control works in the background while infants are cognitively invested in acquiring higher-level skills. This finding is aligned with the results by Mlincek and colleagues (2021) [14] in terms of resources being utilized while performing the sitting skill. Moreover, the fact that greater scores in cognitive, language, and communication behavior are associated with a progressively more repeatable sway pattern in the AP direction and increasing in amplitude in the ML direction, may support the existence of different control mechanisms for the directionality of postural sway during sitting, which has been suggested in the literature [24]. These findings stress the importance of investigating multiple at-risk groups to identify unique developmental pathways that may lead to an ASD diagnosis.

### Limitations

First, a small sample size of the elevated-risk infants was obtained, even though effect sizes were moderate to high. Second, we did not control for birth order in the TD or LBW group, which may have contributed to heterogeneity in those groups. Even though this approach has been adopted by other studies, we need to acknowledge that due to funding limitations and the occurrence of the coronavirus pandemic, no data are available on later group outcomes in terms of ASD diagnosis [3,16]. In addition, our data were collected in the home environment of infants rather than a more tightly controlled laboratory environment. This is especially important for the postural control data since instrumentation noise might influence the results. However, this is a more ecologically valid experimental setup. In addition, most of the utilized questionnaires were based on parental reports rather than direct observation by the researcher, which may lead to biased results.

## 5. Conclusions

Future research with a larger sample size is needed to tease out differences in postural sway data between infants at HR for ASD who will go on and be diagnosed with ASD, based on multiple developmental pathways, such as due to genetics or premature birth. It is also important to include long-term follow-up for diagnosis and other developmental outcomes as well as develop tools that will provide a more sensitive evaluation of the subtle differences among groups. In summary, the examination of sitting posture with subjective and quantitative tools could provide insight into the development of one of the most essential gross motor skills, and how it relates to the progression of other developmental milestones in infants at elevated risk for ASD.

## Figures and Tables

**Table 1:** Participant demographics. Caregivers for all infant participants were parents.

Group	Complications during Pregnancy	Infant’s Weight at Birth (kg)	Mother’s Age at Birth (Years)	Caregiver’s Race/Ethinicity	Infant’s Race/Ethnicity	Caregiver’s Highest Level of Education	Partner’s Highest Level of Education	Household Income (USD)
ASD	40%	3.338 (0.78)	31.2 (4.91)	100% Caucasian	100% Caucasian	40% some college 40% post-graduate 20% 4-year college completion	20% some college 40% post-graduate 40% 4-year college completion	40% USD 100,000–150,000 60% USD 50,000–100,000
LBW	87.5%	1.913 (0.52)	29.1 (4.58)	75% Caucasian 25% African American	75% Caucasian 25% African American	37.5% some college 37.5% 4-year college completion 25% post-graduation	62.5% some college 37.5% 4-year college completion	25% did not respond 62.5% USD 50,000–100,000 12.5% USD 100,000–150,000
TD	25%	3.584 (0.39)	32 (4.36)	87.5% Caucasian 8.3% African American 4.2% Caucasian and Latino	83.3% Caucasian 8.3% African American and Caucasian 4.2% Caucasian and Puerto Rican	66.6% post-graduation 20.8% some college 12.5% 4-year college completion	50% 4-year college completion 25% some college 25% post-graduate	33.3% USD 100,000–150,000 8.3% USD 20,000–50,000 25% USD 50,000–100,000 33.3% over USD 150,000

**Table 2:** Inclusion/Exclusion Criteria.

	*Inclusion Criteria*	*Exclusion Criteria*
**Infants at elevated risk for ASD (familial)**	Younger siblings of children with ASD who met the diagnostic criteria on the Autism Diagnostic Observation Schedule (ADOS), and diagnosis confirmed by two clinical members of the team and SCQ	Born < 37 weeks Birth weight < 2500 gr Birth trauma Head injury Prenatal illicit drug or excessive alcohol exposure Known genetic disorder that would confer increased risk for ASD (e.g., fragile X, or any orthopedic diagnoses
**Infants at elevated risk for ASD (prematurity, LBW)**	Born < 37 weeks Birth weight < 2500 gr	Sibling with ASD, head injury Prenatal illicit drug or excessive alcohol exposure, Known genetic disorder that would confer increased risk for ASD (e.g., fragile X), or any orthopedic diagnoses
**Infants at typical risk for ASD**	Born > 37 weeks Birth weight > 2500 gr	Sibling with ASD, birth trauma, head injury Prenatal illicit drug or excessive alcohol exposure, Known genetic disorder that would confer increased risk for ASD (e.g., fragile X), or any orthopedic diagnoses

**Table 3:** Flow diagram of data collection sessions.

Time Points	Measures
First screening ~4 months	Caregiver perception rating Head circumference SRS, SCQ Parent demographic questionnaire
Onset of sitting 4–5 months	Caregiver perception rating MSEL Eye tracking and sitting posture
1 month post-onset of sitting	Sitting posture and eye tracking
2 months post-onset of sitting	Sitting posture and eye tracking CSBS-DP, communication observation
3 months post-onset of sitting	Sitting posture and eye tracking Head circumference
12 months of age	MSEL, CDIs, CSBS-DP Communication observation Head circumference
18 months of age	MSEL, CDIs, CSBS-DP Head circumference

SRS, Social Responsiveness Scale; SCQ, Social Communication Questionnaire; CSBS-DP, Communication and Symbolic Behavior Scales Developmental Profile; MSEL, Mullen Scales of Early Learning; CDIs, MacArthur-Bates Communicative Development Inventories.

## Data Availability

Not applicable.
